# Reply: Tumour and normal cells differ in the induction and repair of DNA double-strand breaks

**DOI:** 10.1038/sj.bjc.6601573

**Published:** 2004-01-20

**Authors:** J Dahm-Daphi, R A El-Awady, E Dikomey

**Affiliations:** 1 Clinics of Radiotherapy and Radiation Oncology, University Hospital of Hamburg-Eppendorf, Martinistr., Hamburg 52 20246, Germany; 2 Institute of Biophysics and Radiobiology, University Hospital of Hamburg-Eppendorf, Martinistr., Hamburg 52 20246, Germany

## 

**Sir,**

The authors acknowledge the comments advanced by Dr Böhm. A major concern was about the seemingly disparate results presented in our studies (Dikomey *et al*, 1998,^2000^; El-Awady *et al*, 2003). We lastly described (El-Awady *et al*, 2003) that tumour cells significantly differ in the number of radiation-induced dsb. As outlined (El-Awady *et al*, 2003), there is also sufficient evidence for that in the literature. We further found that this difference is correlated with the respective radiosensitivity. In contrast, fibroblasts showed identical numbers of induced dsb and, consequently, survival cannot be correlated with dsb induction (Dikomey *et al*, 1998,^2000^; El-Awady *et al*, 2003). On the other hand, fibroblasts showed different repair capacities, which were then associated with the variation in cellular sensitivity (Dikomey *et al*, 1998,^2000^). No such correlations were found for the tumour strains, and we provided evidence that this is at least partly due to the fact that the measurement of residual dsbs is perturbed by cell cycle effects and apoptosis, which cannot be separated from dsb repair kinetics.

Two points can be extracted from this. (1) There are significant differences between normal and tumour cells in the variation of dsb induction. (2) Standard gel electrophoresis is suitable to determine dsb induction in both tumour and normal cells, but repair capacity can only be determined in normal cells (fibroblasts). This, however, does not mean that the dsb repair capacity is not relevant for the cellular radiosensitivity of tumour cells. To our best knowledge, there are no appropriate methods at hand, for several reasons not even recording of *γ*-H2AX foci, to measure dsb repair in tumour cells.

The differences in dsb induction among tumour cell lines is likely to be explained by variation in their chromatin structure and thus in their protective capacity from DNA damage, while normal cells maintain a constant chromatin structure. This is supported by many recent results emphasising variant patterns of chromatin methylation and histone acetylation in tumour cells. Moreover, the p53 status which is described in a second paper (Böhnke *et al*, 2004) also suggests that p53 could possibly influence the chromatin and hence the amount of induced damage. Those factors may not apply for differentiated normal cells and thus the number of induced DNA damages appears to be fairly constant.

In conclusion, we feel that the data presented are consistent rather than contradictory, as claimed by Dr Böhm. Nevertheless, we agree with the comment that some preceding data should be reviewed again in the light of the recent results. And, secondly, we fully agree that additional factors besides DNA dsb could well (co-)determine the cellular response to radiation, whereby micronuclei and apoptosis, as brought forward by Dr Bohm, may well be the consequence of dsb.
